# Malaria management - progress made and challenges still to face

**DOI:** 10.1186/1475-2875-8-S1-S1

**Published:** 2009-10-12

**Authors:** Bernhards R Ogutu

**Affiliations:** 1 Centre for Clinical Research, Kenya Medical Research Institute, Kisumu, Kenya and Malaria Clinical Trials Alliance-INDEPTH-Network

## 

Although significant advances have been made in the understanding of parasite and vector biology and the treatment and prevention of malaria, *Plasmodium falciparum *continues to inflict a considerable burden of disease, particularly in sub-Saharan Africa, with infants and young children bearing the greatest morbidity and mortality [1].

The introduction of ACT (artemisinin-based combination therapy), especially Coartem^® ^(artemether/lumefantrine; AL), has had a significant impact on the treatment of malaria. A large body of evidence supports consistently high efficacy and safety, demonstrating crucial reliability in vulnerable populations. The efficacy and safety of this ACT have been clearly demonstrated in a clinical development programme spanning 14 years and enrolling approximately 5,000 patients. In trials conducted in different malaria-endemic regions, AL has consistently achieved 28-day PCR-corrected cure rates of >95% in evaluable populations, and has shown a favourable safety and tolerability profile [2-6]. The new dispersible formulation of AL proved as safe and effective as the regular tablets, achieving 28-day PCR-corrected cure rates of >97% [5]. Furthermore, the gametocidal properties of this ACT have been clearly demonstrated, suggesting a favourable impact on malaria transmission [7].

Studies in Zambia and KwaZulu-Natal have demonstrated the impressive impact that AL can have on malaria morbidity and mortality and transmission, at the same time providing cost savings, as presented in this supplement, when used in combination with other malaria control strategies [8-11].

With the increasing availability of generic forms of ACT, it is important to be aware of quality assurance considerations to be sure that the efficacy and safety of these critical anti-malarials is not compromised. Substandard products may contain lower-than-stated doses, or packaging may not be supportive enough to further understanding and adherence to treatment, which could increase the burden of malaria and promote resistance.

Ensuring prompt access to an effective anti-malarial, particularly to remote rural communities in resource-poor countries, is an ongoing challenge. Prescribing health workers and their patients, particularly those who rely on government supplies, continually battle a 'drug is out of stock' syndrome. Rational and implementable anti-malarial drug policies, which are essential decision-support tools in the fight against resistance, are impossible to develop or to implement without an ensured supply of effective, quality assured drugs. Increasingly, community deployment programmes are proving successful in diagnosing and treating malaria patients close to home. The Tigray project, conducted in a remote, rural region of Ethiopia (presented in this supplement [11]) demonstrates how the use of community health workers to deliver prompt, effective treatment can have a significant impact on malaria morbidity and mortality, as well as easing the malaria case load for health facilities [12]. However, this intervention strategy will rely on the individual countries being willing to deregulate ACT to over-the-counter (OTC) drug status.

Diagnostic development represents a potentially powerful strategy to simultaneously improve healthcare delivery and delay emergence of resistance. In the current situation of rapidly evolving malaria epidemiology, accurate diagnosis and appropriate treatment of patients, with both positive and negative results for malaria will be crucial. Informing communities and health care providers that not every fever is malarial in origin and urging them to seek a test before administration of an anti-malarial treatment remains a challenge due to entrenched beliefs and prescribing habits.

Novartis has delivered more than 250 million treatments of AL to malaria-endemic countries on a non-profit basis since 2001, saving an estimated 630,000 lives, and has an on-going commitment to serving vulnerable populations. The recent innovation of a dispersible formulation of AL for infants and young children demonstrates a patient-centric approach - the recognition of an unmet medical need followed by a considered response supported by a rigorous clinical development programme. Infants and young children are most vulnerable to malaria, and the delivery of a formulation specifically designed to meet their needs will hopefully represent a significant advance in the successful treatment of this population.

Patient-centred care will be vital to encourage adherence to new treatment algorithms developed in response to the changing malaria environment in Africa. The proof of efficacy alone is not sufficient - acceptability to both prescribers and their patients is vital. The provision of effective drugs such as AL and other forms of ACT, in appropriate formulations, will encourage improved adherence to drug regimens. Education and communication programmes to engage both healthcare providers and the community will be essential in a new era of malaria prevention and treatment. Ultimately, healthcare delivery systems are crucial to ensure the effectiveness of the medicine, and there needs to be a concerted effort to improve the healthcare systems in Africa, both publicly and privately.

The safety profile of AL in the clinical trials performed so far has remained good. However, there is a need for continued pharmacovigilance to seek any rare untoward effect and the INDEPTH effectiveness and safety studies of anti-malarial drugs in Africa (INESS) will provide the much-needed pharmacovigilance data to supplement the limited spontaneous reporting of adverse events from Africa [13]. The INESS project will help to sensitize and train patients and health care professionals in pharmacovigilance reporting, which is so far limited in Africa through the existing systems in place supported by organizations such as WHO, local health authorities or marketing authorization holders.

This supplement reviews the contribution made by AL as an ACT to the tremendous progress made in the control of malaria and establishes effective treatment as a cornerstone in the foundation of effective, quality patient care for infants, children and adults with malaria.

## Competing interests

The author would like to acknowledge that Novartis Pharma AG sponsored this supplement. However, the author does not work for, or represent in any way, Novartis Pharma AG.

## Authors' contributions

The author met International Committee of Medical Journal Editors criteria for authorship.

## References

[B1] WHO Malaria Factsheet No. 95. Updated January 2009. http://www.who.int/mediacentre/factsheets/fs094/en/index.html.

[B2] van Vugt M, Wilairatana P, Gemperli B, Gathmann I, Phaipun L, Brockman A, Luxemburger C, White NJ, Nosten F, Looareesuwan S (1999). Efficacy of six doses of artemether-lumefantrine (benflumetol) in multidrug-resistant *Plasmodium falciparum *malaria. Am J Trop Med Hyg.

[B3] van Vugt M, Looareesuwan S, Wilairatana P, McGready R, Villegas L, Gathmann I, Mull R, Brockman A, White NJ, Nosten F (2000). Artemether-lumefantrine for the treatment of multidrug-resistant falciparum malaria. Trans R Soc Trop Med Hyg.

[B4] Lefèvre G, Looareesuwan S, Treeprasertsuk S, Krudsood S, Silachamroon U, Gathmann I, Mull R, Bakshi R (2001). A clinical and pharmacokinetic trial of six doses of artemether-lumefantrine for multidrug-resistant *Plasmodium falciparum *malaria in Thailand. Am J Trop Med Hyg.

[B5] Abdulla S, Sagara I, Borrmann S, D'Alessandro U, González R, Hamel M, Ogutu B, Mårtensson A, Lyimo J, Maiga H, Sasi P, Nahum A, Bassat Q, Juma E, Otieno L, Björkman A, Beck HP, Andriano K, Cousin M, Lefèvre G, Ubben D, Premji Z (2008). Efficacy and safety of artemether-lumefantrine dispersible tablets compared with crushed commercial tablets in African infants and children with uncomplicated malaria: a randomised, single-blind, multicentre trial. Lancet.

[B6] Hatz C, Soto J, Nothdurft HD, Zoller T, Weitzel T, Loutan L, Bricaire F, Gay F, Burchard GD, Andriano K, Lefèvre G, De Palacios PI, Genton B (2008). Treatment of acute uncomplicated falciparum malaria with artemether-lumefantrine in non-immune populations: a safety, efficacy, and pharmacokinetic study. Am J Trop Med Hyg.

[B7] Sutherland CJ, Ord R, Dunyo S, Jawara M, Drakeley CJ, Alexander N, Coleman R, Pinder M, Walraven G, Targett GA (2005). Reduction of malaria transmission to Anopheles mosquitoes with a six-dose regimen of co-artemether. PLoS Med.

[B8] Chizema-Kawesha E, Mukonka V, Mwanza M, Kaliki C, Phiri M, Miller J, Komatsu R, Aregawi M, Masaninga F, Kitikiti S, Babaniyi O, Otten M Evidence of substantial nationwide reduction of malaria cases and deaths due to scale-up of malaria control activities in Zambia, 2001-2008. World Health Organization, Zambia 19-23 January Impact Evaluation Mission Report.

[B9] Chanda P, Masiye F, Chitah BM, Sipilanyambe N, Hawela M, Banda P, Okorosobo T (2007). A cost-effectiveness analysis of artemether lumefantrine for treatment of uncomplicated malaria in Zambia. Malar J.

[B10] Barnes KI, Durrheim DN, Little F (2005). Effect of artemether-lumefantrine policy and improved vector control on malaria burden in KwaZulu-Natal, South Africa. PLoS Med.

[B11] Barnes K, Chanda P, Barnabas GA (2009). Impact of the large-scale deployment of artemether/lumefantrine on the malaria disease burden in Africa: case studies of South Africa, Zambia and Ethiopia. Malar J.

[B12] Lemma H, Desta A, Fottrell E, Barnabas GA, Bianchi A, Bosman A, Byass P, Costanzo G, Morrone A, Mulure N, Toma L (2008). Community-level deployment of artemether lumefantrine (AL) with rapid diagnostic testing: effect on malaria outcomes and resource utilization in a rural setting [abstract]. Am J Trop Med Hyg.

[B13] INDEPTH Network. http://www.indepth-network.org/.

